# Understanding Well-Dying Research in a Rapidly Aging Society: A Text Network and Topic Modeling Analysis of Korean Academic Publications

**DOI:** 10.3390/healthcare14172768

**Published:** 2026-09-01

**Authors:** Jin-Hui Ku, Kwang-Hwan Kim

**Affiliations:** 1 Division of Software Liberal Arts, University of Mokwon, Daejeon 35349, Republic of Korea; jhku@mokwon.ac.kr; 2 Well-Dying Convergence Laboratory, University of Konyang, Daejeon 35365, Republic of Korea

**Keywords:** well-dying, rapid population aging, super-aged society, text network analysis, topic modeling, life-sustaining treatment

## Abstract

**Highlights:**

**What are the main findings?**

- We identified four major research domains in well-dying studies via text network analysis and LDA topic modeling: psychological well-being and death preparation, social and policy approaches, healthcare education, and legal–ethical decision-making.
- Well-dying research has evolved from individual-level death awareness toward broader discussions encompassing healthcare systems, community-based support, public policy, and life-sustaining treatment decisions.

**What are the implications of the main findings?**

- The findings demonstrate that well-dying is increasingly recognized as a multidimensional social and healthcare issue requiring integrated approaches across the medical, welfare, legal, and community sectors.
- As one of the world’s fastest-aging societies, the Korean population provides valuable insights for countries preparing for similar demographic transitions and the development of sustainable end-of-life care systems.

**Abstract:**

**Background/Objectives**: Population aging has emerged as a major global challenge, particularly in Asian countries experiencing rapid demographic transitions. Among them, South Korea represents one of the fastest aging societies in the world, having rapidly transitioned into a super-aged society. As aging populations expand worldwide, increasing attention has been directed toward well-dying as an important component of quality of life, end-of-life care, and social well-being in later life. This study aims to identify the major research trends and knowledge structures of well-dying studies by applying text net-work analysis and LDA-based topic modeling. **Methods**: A total of 91 Korean academic studies related to well-dying published between 2016 and March 2026 were collected from publicly accessible scholarly databases and analyzed via keyword frequency, degree centrality, and community detection analyses, as well as LDA-based topic modeling. **Results**: The results showed that keywords such as “death,” “awareness,” “education,” “older adults,” “medical care,” and “life-sustaining treatment” played central roles in the knowledge network. Community analysis revealed that well-dying research has evolved into interconnected domains involving psychological preparations for death, hospice and palliative care, legal and ethical decision-making, and community-based aging policies. Topic modeling further identified four major themes: (1) psychological well-being and death preparation in later life, (2) social and policy approaches to well-dying, (3) well-dying education and healthcare perceptions, and (4) legal and ethical issues surrounding life-sustaining treatment decisions. **Conclusions**: The findings suggest that well-dying research is expanding from individual psychological adaptation to broader social, medical, legal, and policy dimensions. As one of the world’s fastest-aging societies, the Korean case provides meaningful implications for other countries facing accelerated population aging and highlights the importance of integrated well-dying policies and community-based support systems in super-aged societies.

## 1. Introduction

Population aging has become one of the most significant demographic transformations worldwide. According to the United Nations, the global population aged 65 years and older is projected to increase more than twofold by 2050, reaching approximately 1.6 billion people worldwide [1]. In particular, Asian countries are experiencing demographic transitions at an unprecedented speed due to declining fertility rates and increased life expectancy. As societies rapidly transition from aging to super-aged populations, concerns regarding quality of life, end-of-life care, and dignified death have emerged as major public health and social policy issues [2].

Among rapidly aging countries, South Korea represents one of the fastest transitions to a super-aged society in the world, becoming an aged society in 2017 and surpassing the threshold of a super-aged society, with more than 20% of its population aged 65 years or older, in less than a decade. This demographic transformation is occurring much faster than previously experienced in many Western countries or Japan, generating substantial challenges in healthcare systems, long-term care, social welfare, family structures, and end-of-life decision-making. Consequently, South Korea provides an important early case for understanding how societies respond to accelerated aging and how well-dying research evolves within super-aged contexts. Rapid population aging has transformed not only demographic structures but also family relationships, care systems, healthcare delivery, and social welfare policies. Declining fertility, increasing life expectancy, and the rise of single-person older households have shifted end-of-life care from a predominantly family responsibility toward institutional and community-based support systems.

Well-dying refers not merely to the preparation for death but to the process of achieving a meaningful and dignified end of life based on self-determination, human dignity, psychological well-being, and social connectedness. International studies have often discussed well-dying within the broader concept of a “good death,” emphasizing multidimensional factors such as physical comfort, emotional stability, interpersonal relationships, autonomy, and spiritual integration [3,4]. Furthermore, hospice and palliative care have been recognized as essential approaches for improving quality of life at the end of life and reducing unnecessary suffering among patients and families [5]. These demographic and social transformations have also reshaped the conceptual understanding of well-dying. As responsibilities for end-of-life care have gradually shifted from families to healthcare systems, community organizations, and public institutions, well-dying has increasingly been recognized not only as an individual’s preparation for death but also as a multidimensional societal issue requiring coordinated medical, psychosocial, legal, and policy responses. Consequently, well-dying is no longer understood solely as achieving a peaceful death but as maintaining dignity, autonomy, social participation, and quality of life throughout the later stages of the life course.

In parallel with rapid aging, interest in well-dying has expanded significantly in South Korea. In particular, following the enactment of the Act on Hospice and Palliative Care and Decisions on Life-Sustaining Treatment for Patients in the Dying Process in 2016 [6], public and academic discussions regarding life-sustaining treatment decisions, advance directives, patient autonomy, and dignified death intensified [7]. Since then, research related to death awareness, death preparation education, hospice care, psychological well-being, self-determination, and aging policy has rapidly increased across multiple disciplines, including nursing, social welfare, psychology, healthcare, and public policy.

Despite the growing body of literature, previous studies on well-dying have largely focused on specific topics or individual empirical cases. Although previous studies have examined well-dying from clinical, psychological, ethical, or educational perspectives, relatively little attention has been paid to how the knowledge structure of well-dying research itself has evolved in response to demographic transition and institutional change. In particular, the period from 2016 to 2026 represents a critical transitional phase in Korea, during which demographic aging accelerated alongside institutional reforms, changing social attitudes toward death, and expanding policy discussions regarding end-of-life care. Understanding how well-dying research evolved during this period may provide meaningful insights not only for Korea but also for other countries facing similar demographic transitions.

Therefore, this study aims to identify the major research trends and knowledge structures of well-dying studies in a rapidly aging society through text network analysis and topic modeling. To achieve this purpose, Korean academic studies related to well-dying published between 2016 and March 2026 were collected from publicly accessible scholarly databases and analyzed. Specifically, this study examines keyword frequencies, network centrality, community structures, and latent research topics to understand how well-dying research has evolved in response to accelerated population aging and changing end-of-life care environments. By shifting the analytical focus from a simple descriptive overview to a structural and multi-dimensional comprehension of the academic discourse, this study addresses the following three research questions:

**RQ** **1.** *What are the major research trends of well-dying research in Korea?*

**RQ** **2.** *What structural knowledge networks are formed among the major research topics, and how do they reflect institutional or societal shifts?*

**RQ** **3.** *What latent thematic structures characterize the evolving academic discourse on well-dying, and how do these themes complement the knowledge network identified through text network analysis?*

The findings are expected to provide important implications for future well-dying research, aging policies, hospice and palliative care systems, and community-based support strategies in super-aged societies.

**Figure 1:**
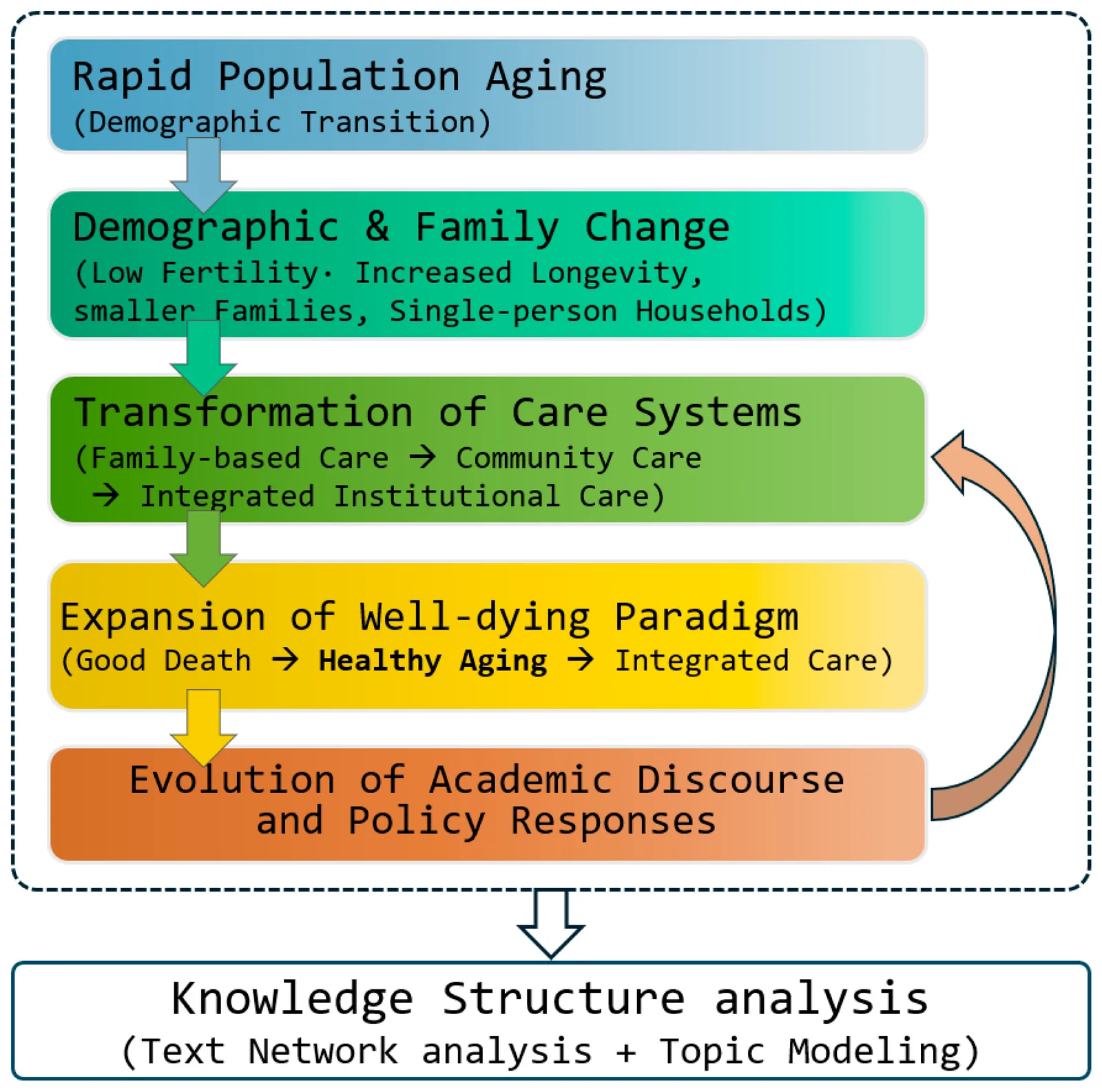
Conceptual framework illustrating how rapid population aging drives demographic and institutional changes, reshapes the well-dying paradigm, and ultimately influences the evolution of academic discourse, which in turn contributes to policy responses and further transformations of care systems.

Figure 1 illustrates the conceptual framework underpinning this study. As rapid population aging reshapes family structures and care systems, the concept of well-dying has expanded from an individual concern about a “good death” to a broader paradigm encompassing healthy aging, integrated care, and societal responsibility. Based on this framework, this study examines how the academic discourse on well-dying has evolved by analyzing its knowledge structure through text network analysis and LDA topic modeling. This framework assumes that demographic transition influences changes in family structures and care systems, which in turn reshape the conceptualization of well-dying and ultimately influence the evolution of academic discourse. Importantly, this process is not linear. Rather, evolving academic discourse also contributes to policy responses and institutional improvements, which further transform care systems in an iterative manner.

## 2. Materials and Methods

### 2.1. Research Design

This study was guided by the conceptual framework presented in Figure 1, which explains how rapid population aging drives demographic and family changes, transforms care systems, expands the conceptualization of well-dying, and ultimately influences the evolution of academic discourse. Based on this framework, this study employed text network analysis and Latent Dirichlet Allocation (LDA) topic modeling to investigate how the knowledge structure of well-dying research has evolved in response to demographic transition and institutional change in South Korea.

Unlike conventional literature reviews that primarily summarize previous findings, text network analysis enables the systematic identification of semantic relationships among research concepts and the structural characteristics of an academic field. In the present study, keyword frequency analysis, co-occurrence analysis, degree centrality analysis, and community modularity analysis were performed to identify the major research domains, conceptual relationships, and structural characteristics of well-dying research.

To complement the structural perspective provided by text network analysis, LDA topic modeling was employed to identify latent thematic structures embedded within the collected literature. While text network analysis reveals explicit semantic relationships among keywords, LDA identifies hidden thematic patterns that may not be directly observable through network structures alone. The complementary application of these two analytical approaches provides a more comprehensive understanding of the evolution of well-dying research.

Accordingly, this study integrates text network analysis and topic modeling to examine not only the structural relationships among major research concepts but also the latent thematic evolution of well-dying research within the broader context of rapid population aging and changing end-of-life care systems.

### 2.2. Data Collection and Preprocessing

A total of 91 peer-reviewed Korean academic articles related to well-dying published between January 2016 and March 2026 were collected for analysis. The literature search was conducted using the Korean Citation Index (KCI; https://www.kci.go.kr/), the largest national academic database indexing peer-reviewed scholarly journals published in South Korea. Since the objective of this study was to investigate the evolution of well-dying research within the Korean academic context, KCI was considered the most appropriate and comprehensive source for identifying relevant literature.

The literature retrieval was completed on 1 April 2026. The study period (January 2016–March 2026) was selected because it represents a decade of substantial demographic transition in South Korea, during which the country rapidly progressed toward a super-aged society and experienced important institutional and social changes related to end-of-life care and aging policy.

To ensure that the retrieved publications focused primarily on well-dying rather than merely mentioning the concept, the search strategy required the Korean title to contain the term “웰다잉” (well-dying) and the English author keywords to include “well-dying.” Publications were retrieved using KCI’s advanced search function. Duplicate records and publications outside the scope of peer-reviewed KCI-indexed journal articles were excluded during the screening process. Consequently, 91 articles satisfied the inclusion criteria and constituted the final dataset for analysis.

Bibliographic information, including the title, authors, journal, publication year, abstract, and author keywords, was collected for subsequent analysis. The detailed literature search strategy is summarized in Table A1 (Appendix A).

For text preprocessing, titles and abstracts were selected as the primary textual data, and natural language preprocessing, including tokenization and noun extraction, was conducted using Python libraries such as NLTK v3.9.1 and KoNLPy v0.6.0. Since Korean language processing often involves spacing inconsistencies and linguistic variations, repeated preprocessing and manual refinement were performed to improve semantic consistency.

Stopwords, including overly generic academic terms such as research, analysis, and results, were removed from the dataset. In addition, synonymous or semantically equivalent terms were manually standardized. For example, terms related to legal systems (e.g., law, legislation, and act) and gender-related expressions were unified during preprocessing, while single-character Korean words and semantically ambiguous terms were excluded. Repeated manual verification was also performed throughout the preprocessing stage to improve semantic consistency and analytical reliability.

All preprocessing procedures and subsequent analyses were conducted using Microsoft Excel LTSC 2024, Python (NLTK v3.9.1, KoNLPy v0.6.0, Gensim v4.4.0, and pyLDAvis v3.4.1), and NetMiner v4.5.

### 2.3. Keyword Selection Using TF-IDF

To identify core keywords representing the major themes of well-dying research, TF-IDF (Term Frequency–Inverse Document Frequency) analysis was performed. TF-IDF is a statistical method used to evaluate word importances within a collection of documents by considering both the frequency of a word within a document and its rarity across the entire corpus.

(1) $$TF-IDF=tf\left(d,t\right)\times idf(d,t)\;\;idf(d,t)=\log\left(\frac{n}{1+df(t)}\right)\;$$

Equation (1) presents the TF-IDF calculation formula (d: document, t: word, and n: total number of documents). Additionally, TF (term frequency) refers to the number of times a specific word t appears in a specific document d, and DF (document frequency) refers to the number of documents in which the specific word t appears, with IDF as the reciprocal of the DF value.

Words with high term frequencies generally represent important concepts within a document, whereas TF-IDF further evaluates the discriminative importance of those terms across the entire corpus. In this study, the 131 core keywords were selected using predefined frequency-based criteria (minimum term frequency = 10 and minimum word length = two characters). Therefore, keyword inclusion was determined by frequency thresholds rather than TF-IDF values.

After the core keywords had been selected, TF-IDF was calculated to evaluate the discriminative importance of the selected keywords across the corpus. The average TF-IDF weight of the 131 selected keywords was 0.447, indicating that the selected keywords were sufficiently informative for representing the semantic characteristics of the corpus.

### 2.4. Co-Occurrence Analysis

Co-occurrence analysis identifies semantic relationships among keywords based on the frequency with which two or more keywords appear together within the same document. In this study, each academic article was treated as a single analysis unit, and co-occurrence relationships were calculated at the document level.

(2) $$Co-occurrence\left(A,\;B\right)=\sum_{i=1}^{N}I(A\in D_{i}\;\wedge \;B\in D_{i})$$

Equation (2) represents the co-occurrence frequency between keywords A and B (N: number of total documents (or analysis units), $D_{i}:$ i-th document, and $I\left(\;\right):$ indicator function that returns 1 if A and B exist simultaneously in $D_{i}$ and 0 otherwise).

Co-occurrence analysis enables the identification of semantic connectivity and conceptual relationships among keywords beyond simple frequency analysis [8,9]. Since highly frequent keywords may produce overly complex network structures, the former was used to clarify major topic structures and relationships among concepts within well-dying research.

### 2.5. Structuring and Visualizing Text Networks

#### 2.5.1. Degree Centrality and Community Modularity

Degree centrality analysis was conducted to identify keywords that play central roles within the semantic network. Degree centrality indicates the extent to which a keyword is connected to other keywords and is commonly used to identify influential concepts within a network.

(3) $$Degree\;Centrality\;C_{D}\left(i\right)=\sum_{j\in N,\;j\neq i}W_{ij}$$

Equation (3) represents the degree centrality of node i. It is calculated as the total number of times node i appears together with other nodes (j) within a single sentence (or unit of analysis).

In addition, community modularity analysis was performed using the Clauset–Newman–Moore (CNM) algorithm to identify sub-network structures within the keyword network. The CNM algorithm is a hierarchical clustering method that detects communities by maximizing modularity values and is suitable for identifying interpretable semantic clusters in small- to medium-sized networks.

(4) $$Q=\frac{1}{2m}\sum_{i,j}(A_{ij}-\frac{k_{i}k_{j}}{2m})\delta (c_{i},\;c_{j})$$

Equation (4) represents the modularity function used to evaluate community structures within the network (m: total number of edges, $A_{ij}$: adjacency matrix, $k_{i}$: degree of node i, and $\delta (c_{i},\;c_{j})$: returns 1 if node i and node j belong to the same community and 0 otherwise).

Modularity values between 0.3 and 0.7 are generally interpreted as indicating meaningful community structures [10,11], and the CNM algorithm was selected because it enables semantic clusters and conceptual subtopics to be intuitively interpreted within well-dying research.

#### 2.5.2. LDA Topic Modeling

Topic modeling was conducted to identify latent thematic structures within the collected documents. Among topic modeling approaches, Latent Dirichlet Allocation (LDA) is one of the most widely used probabilistic methods for discovering hidden topics in large-scale textual data [12,13].

LDA assumes that documents consist of multiple latent topics and that each topic is represented by a distribution of keywords. Compared with earlier methods such as Latent Semantic Indexing (LSI) and probabilistic Latent Semantic Indexing (pLSI), LDA provides improved interpretability and extensibility for semantic analysis.

The most important step in LDA topic modeling is determining the optimal number of topics. If too few topics are selected, overly broad themes may emerge; conversely, too many topics may produce fragmented or redundant clusters. Therefore, topic coherence was used to evaluate semantic interpretability among topic keywords and to determine the optimal number of topics [14,15,16].

(5) $$Coherence\;\left(W\right)=\sum_{w_{1},w_{2}\;\in \;W}\log\frac{D\left(w_{1},w_{2}\right)+\epsilon }{D(w_{2})}$$

In Equation (5), $D(w_{1},\;w_{2})$ represents the frequency of documents in which two words $w_{1}$ and $w_{2}$ appear simultaneously in the entire corpus, while $D(w_{2})$ represents the frequency of documents in which word $w_{2}$ appears simultaneously in the entire corpus, and ε is a smoothing factor added to prevent the value inside the denominator or logarithm from becoming zero [17].

Higher coherence values indicate stronger semantic relationships among keywords within a topic and therefore greater interpretability of the topic structure. In this study, the final number of topics was determined by repeatedly comparing coherence scores and evaluating topic interpretability through visualization using pyLDAvis v3.4.1.

## 3. Results

### 3.1. Keyword Frequency Analysis

In this study, a text mining-based keyword frequency analysis was conducted on 91 well-dying-related academic publications published between 2016 and March 2026 and retrieved from publicly accessible Korean scholarly databases. Term frequency (TF) represents how frequently a specific word appears within a collection of documents and is widely used to identify major themes and research interests within a research field.

To improve the reliability of the trend analysis, core keywords were selected based on a minimum word length of two characters and a minimum term frequency of 10. As a result, a total of 131 core keywords were extracted. Among them, the top 30 keywords showed frequencies of 30 or higher. Table 1 presents the top 30 most frequently occurring keywords and their frequencies.

The analysis revealed that the top 30 keywords appeared more than 30 times each, indicating that several major themes have been consistently and intensively discussed within well-dying research. Among them, “death” showed the highest frequency (351 occurrences), confirming its role as the central concept in well-dying discourse. This was followed by keywords such as “awareness,” “education,” “older adults,” and “society,” suggesting that well-dying research has expanded beyond simple discussions of death toward multidimensional discussions involving death awareness, death preparation education, and aging-related quality of life.

In particular, keywords such as “life-sustaining treatment,” “withdrawal,” “patient,” “decision,” “law,” “ethics,” and “advance directives” indicate that research attention has increasingly focused on end-of-life decision-making and patient self-determination following the implementation of legal and institutional frameworks regarding life-sustaining treatment decisions. These findings suggest that well-dying research has evolved beyond psychological preparations for death into broader discussions involving healthcare practice, legal legitimacy, patient rights, and ethical decision-making.

Furthermore, keywords such as “hospice,” “nursing,” “medical care,” “psychological well-being,” “welfare,” “community,” “culture,” and “policy” demonstrate that well-dying research is increasingly connected to hospice and palliative care, emotional support, community-based care systems, and social welfare policies. In particular, studies emphasizing psychological stability, quality of life, emotional acceptance of death, and dignity at the end of life indicate that well-dying is increasingly understood as an integrated concept associated with successful aging and holistic end-of-life care.

The expanded analysis of the top 50 keywords additionally identified terms such as “anxiety,” “well-being,” “emotion,” “reflection,” and “later life,” suggesting that psychological and emotional dimensions have become major research domains in well-dying studies. Additionally, research related to death anxiety reduction, emotional adaptation, self-reflection, and meaning-making in later life has been actively conducted.

Overall, the findings suggest that well-dying research in a rapidly aging society has evolved from early studies focused on death awareness and psychological acceptance toward more integrated discussions involving healthcare, law, ethics, community care, welfare systems, and public policy. These results indicate that well-dying is increasingly recognized not merely as an individual issue, but as a multidimensional social agenda closely associated with quality of life, dignity, and integrated support systems in super-aged societies.

### 3.2. Degree Centrality and Community Modularity Analysis

#### 3.2.1. Degree Centrality of Core Keywords

Degree centrality analysis was conducted to identify the connectivity structure and influential concepts within the well-dying keyword network. Degree centrality represents the extent to which a keyword is connected to other keywords and is commonly used to identify central concepts within semantic networks.

Table 2 shows the degree centrality scores of the 131 keywords and the top 30 keywords. The analysis of 131 core keywords revealed an average degree centrality of 0.597, a standard deviation of 0.189, and a minimum value of 0.046. The Network Degree Centralization Index was calculated as 40.93%, indicating that the overall keyword network formed a relatively concentrated structure centered around several influential concepts.

In contrast, the network of the top 30 keywords showed a lower centralization index (11.084%), indicating that major keywords formed a relatively balanced and interconnected semantic structure rather than relying excessively on a small number of dominant nodes.

**Figure 2:**
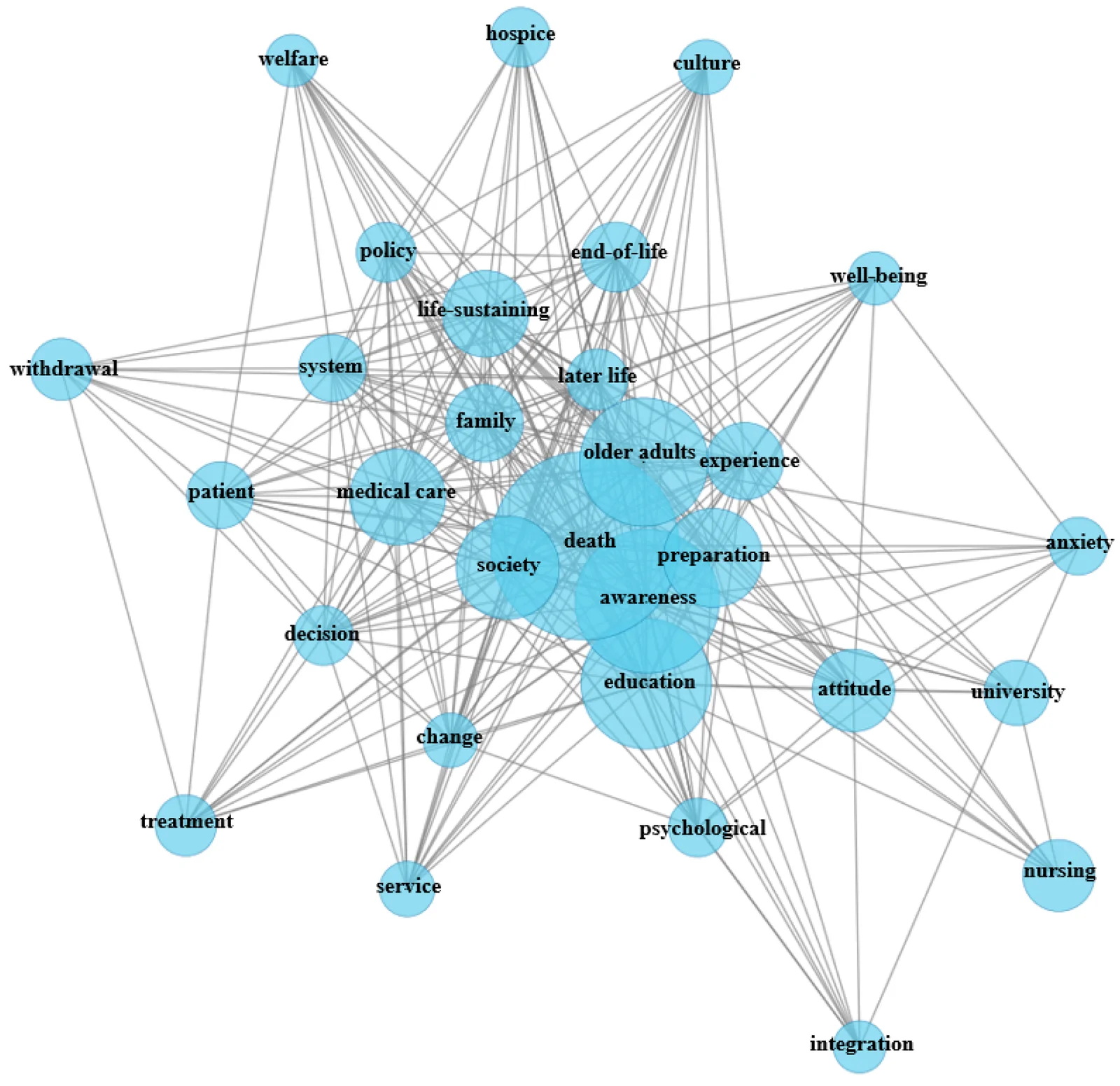
Network visualization of the top 30 core keywords.

Figure 2 presents the network visualization results for the top 30 keywords. To improve interpretability and reduce excessive network complexity, the visualization was limited to the top 30 high-frequency keywords. Additionally, co-occurrence relationships were calculated at the document level, and the network graph was generated based on a minimum co-occurrence frequency of three or higher. The network consisted of 30 nodes and 276 edges, where node size was proportional to keyword frequency, whereas edge thickness represented co-occurrence frequency.

The visualization revealed that keywords such as “death,” “awareness,” “education,” “older adults,” “preparation,” and “society” occupied central positions within the network and demonstrated strong connectivity with various other concepts. Meanwhile, keywords such as “hospice,” “psychological well-being,” “welfare,” “culture,” and “nursing” were relatively peripheral but remained closely linked to the central concepts. These findings indicate that well-dying research is expanding into medical, welfare, cultural, and psychological domains while maintaining a core semantic structure centered on death awareness, preparation, and aging.

Overall, the network structure demonstrates that well-dying research in a rapidly aging society is evolving into a convergent research domain in which psychological adaptation, death preparation education, hospice and palliative care, legal decision-making, and community support systems are organically interconnected.

Table 3 shows the degree centrality scores and community analysis results of the top 20% of core keywords among 131 keywords. Among these, those numbered 1, 2, and 12–19 are the top 10% of words in terms of frequency.

These results suggest that well-dying research is forming a multi-layered structure that starts with an individual’s acceptance of death and psychological preparation, and gradually expands into medicine, welfare, policy, and culture domains. Furthermore, it is judged that future well-dying research will become increasingly important for studies that integrally consider not only medical and legal/institutional approaches but also community-based care, psychological well-being, and life-course perspectives.

#### 3.2.2. Co-Occurrence-Based Degree Centrality and Community Modularity

**Figure 3:**
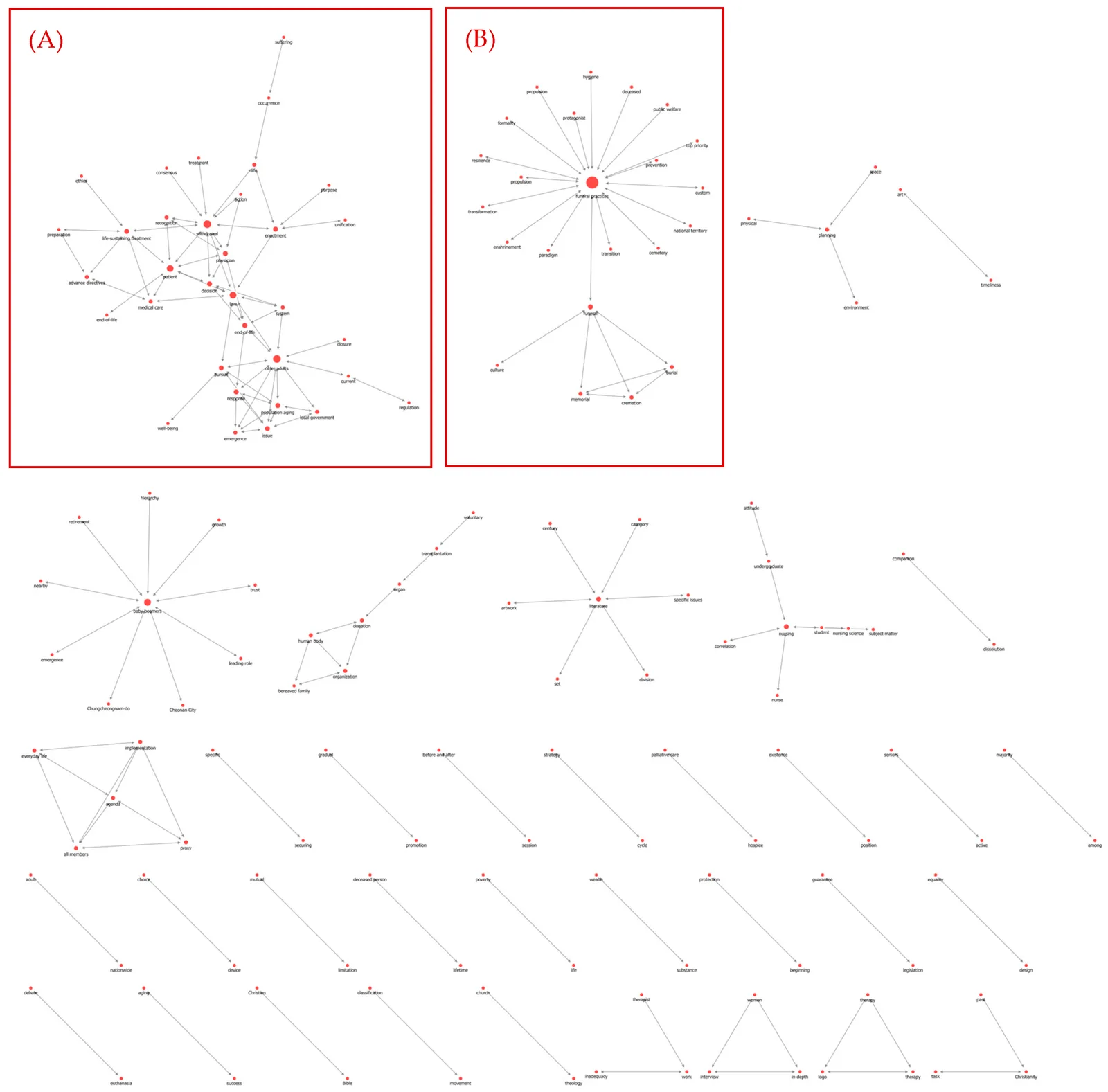
Co-occurrence-based degree centrality network. (A) Dominant network group selected for further community modularity analysis and presented in Figure 4; (B) dominant network group selected for further community modularity analysis and presented in Figure 5.

In text network analysis, if there are many keywords (nodes), the connected edges become entangled, making the network complex and interpretation of the visualization results difficult. Therefore, as shown in Figure 3, degree centrality analysis is generally performed on a small number of high-frequency keywords. In such cases, since only the number of connections is counted, it is impossible to determine how influential or reliable the relationships are. This is because even if centrality is high, there may be limitations in actually spreading information or influence. Furthermore, since other paths that do not pass through the central node are not considered at all when information is transmitted within the network, it is difficult to grasp the overall flow or structural positioning between nodes.

Figure 3 visualizes degree centrality based on co-occurrence frequency—that is, which keywords have been frequently studied together—for all keywords, rather than limited keywords above a certain frequency. Figure 3 (A), (B) represent the dominant semantic clusters within the network.

Community modularity analysis based on the Clauset–Newman–Moore (CNM) algorithm was conducted, identifying groups of keywords that are densely connected within a network, thereby making it useful for detecting conceptual subthemes and latent knowledge structures.

**Figure 4:**
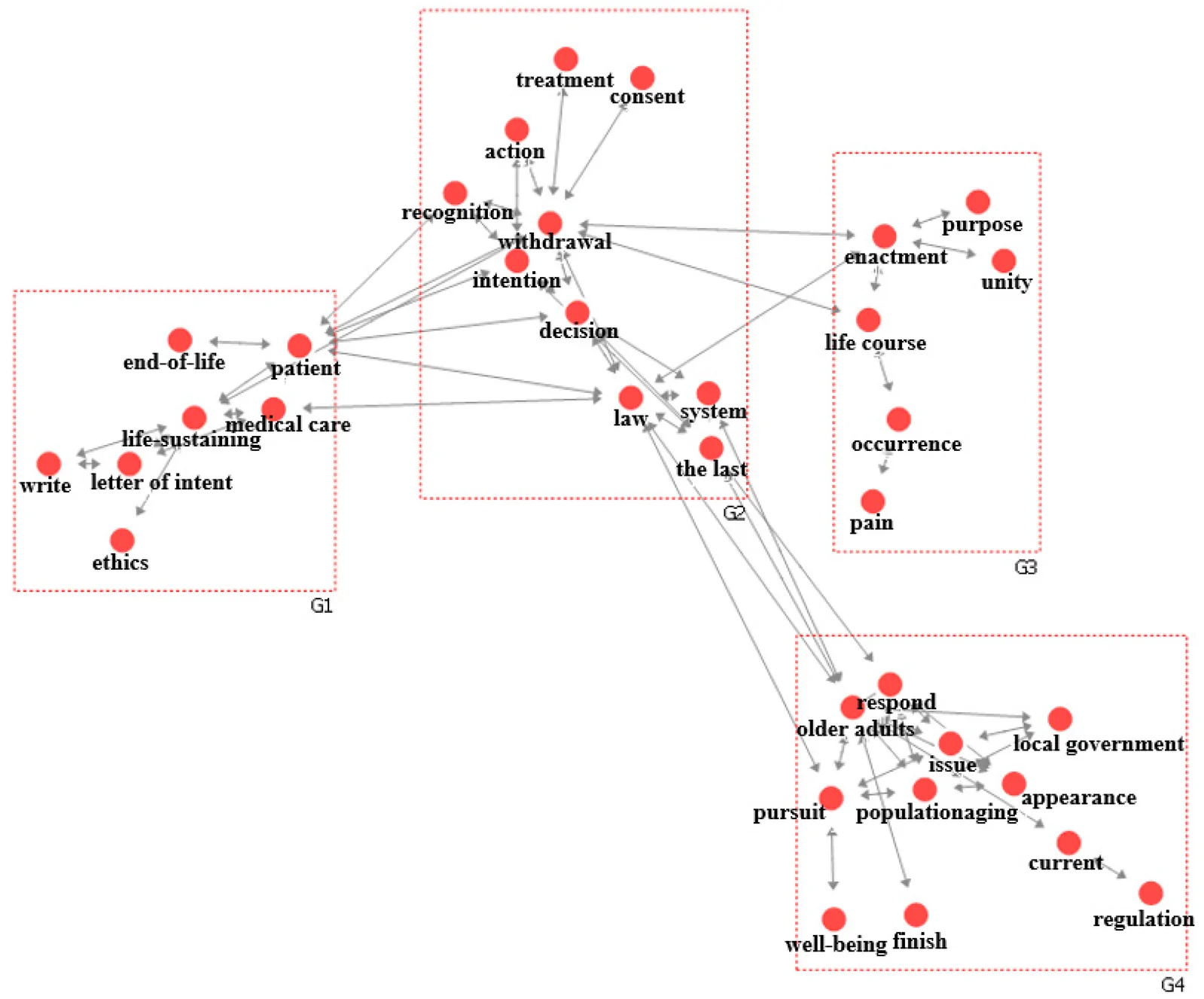
Community modularity structure of core keywords (Figure 3 (A)).

As a result of the major keyword network analysis for (A), four major communities (G1–G4) with an optimal modularity value of 0.473 were identified (Figure 4). Since modularity values above 0.3 generally indicate meaningful community structures, the results suggest that well-dying research forms relatively distinct and interpretable thematic clusters.

- G1: Life-Sustaining Treatment and Patient Self-Determination

G1 consisted of keywords such as “patient,” “medical care,” “life-sustaining treatment,” “advance directives,” “writing,” and “ethics.” This cluster reflects research related to end-of-life decision-making in clinical settings, particularly issues involving patient autonomy, advance care planning, and ethical decision-making. The directional structure of the network demonstrated strong connections between “life-sustaining treatment,” “medical care,” “advance directives,” and “ethics,” suggesting that discussions regarding patient self-determination and dignity have become central themes within well-dying research.

- G2: Legal and Institutional Decision-Making

G2 included keywords such as “treatment,” “withdrawal,” “decision,” “law,” “institution,” and “consent.” This cluster reflects legal and institutional discussions regarding the withdrawal of life-sustaining treatment and medical decision-making processes. The directional flow within the network suggested that medical judgment and patient intention were closely connected to institutional approval and legal frameworks, indicating that end-of-life decision-making has increasingly become a major legal and policy issue in rapidly aging societies.

- G3: Human Dignity and Pain Alleviation

G3 was composed of keywords such as “life course,” “pain,” “integration,” and “purpose.” This cluster reflects research emphasizing human dignity, meaning-making, and pain alleviation during the dying process. The semantic structure indicates that well-dying research is evolving beyond simple death preparation toward integrated approaches that emphasize quality of life, emotional acceptance, and the preservation of dignity at the end of life.

- G4: Responses to a Super-Aged Society and Community-Based Care

G4 consisted of keywords such as “aging,” “well-being,” “community,” “local government,” “policy,” and “regulation.” This cluster reflects growing research interest in community-based support systems and policy responses to super-aged societies. The network’s directionality demonstrated strong connections between “aging,” “policy,” “community,” and “well-being,” indicating that well-dying research increasingly emphasizes integrated support systems involving public policy, local communities, and welfare services.

Overall, the community analysis of (A) revealed that well-dying research forms a multidimensional structure involving healthcare, law, ethics, human dignity, and community-based policy systems. These findings suggest that well-dying research is expanding from individual-level psychological adaptation toward broader institutional and societal dimensions.

**Figure 5:**
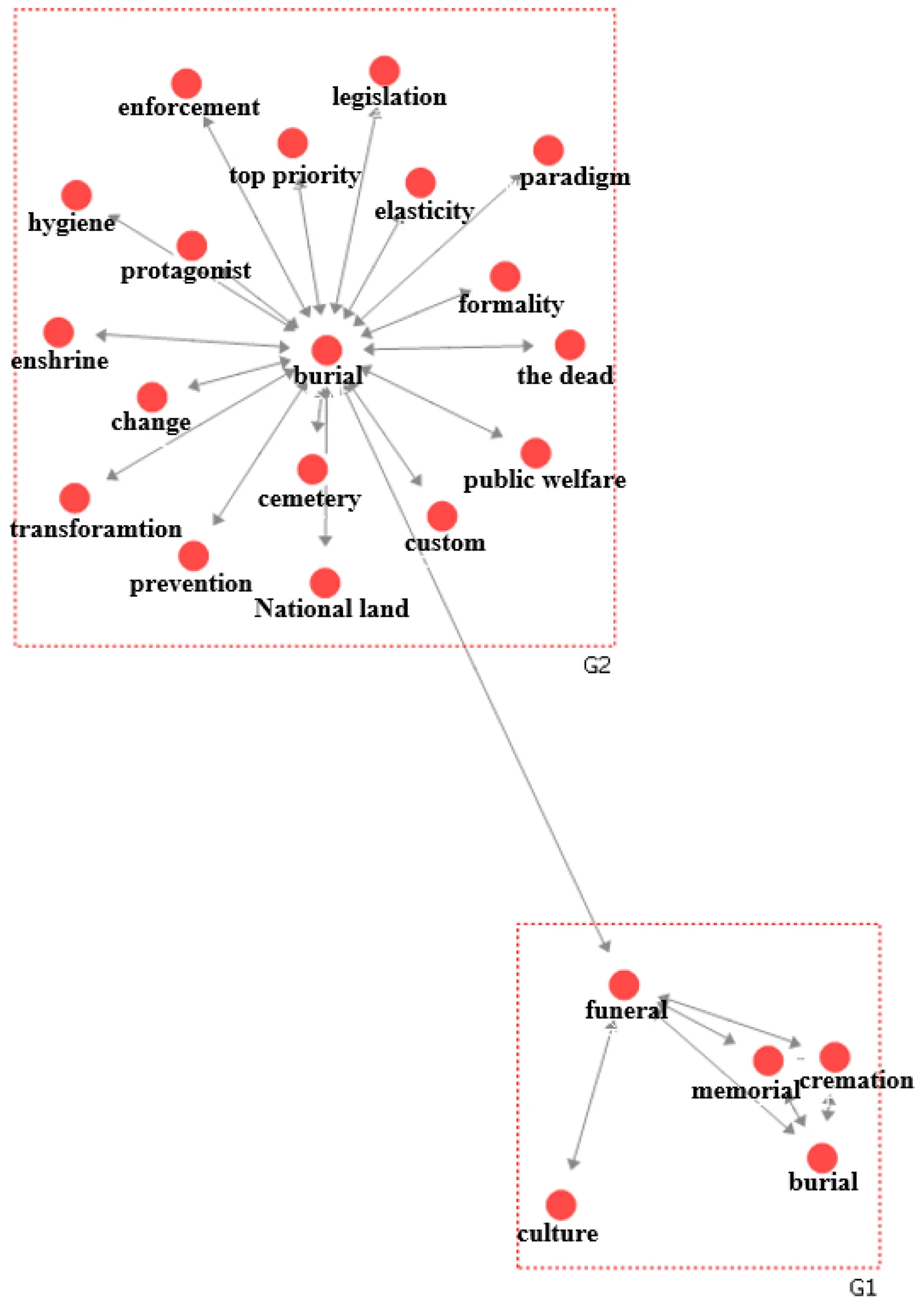
Community modularity structure of core keywords (Figure 3 (B)).

In addition, in the modularity analysis of (B), which is related to funeral and memorial culture, two meaningful communities with an optimal modularity value of 0.38 were identified (Figure 5). The results demonstrated that funeral systems, memorial practices, cremation, and eco-friendly funeral culture are emerging as independent research themes within well-dying studies. This finding suggests that well-dying research is increasingly extending beyond pre-death care to include socio-cultural processes following death.

- G1: Cluster Centered on Funeral Culture and Memorial Methods

G1 was composed of keywords such as ‘funeral,’ ‘memorial,’ ‘burial,’ ‘cremation,’ and ‘culture.’ This cluster is interpreted as being formed around research related to funeral culture, memorial methods, and changes in burial culture.

In particular, a directional trend emerged connecting ‘funeral’ to ‘memorial,’ ‘burial,’ and ‘cremation,’ indicating that post-death rituals and modes of remembrance are being treated as important research topics in well-dying studies. Furthermore, the connection with ‘culture’ suggests that funeral practices are not merely processing procedures but are also closely linked to social and cultural values.

- G2: Cluster Centered on Funeral Systems and Public Policy

G2 was composed of keywords such as ‘burial,’ ‘legislation,’ ‘public welfare,’ ‘national land,’ ‘custom,’ ‘hygiene,’ ‘prevention,’ ‘change,’ and ‘paradigm.’ This cluster is interpreted as having been formed around institutional approaches related to funeral systems, public policy, and changes in funeral culture. Examining the network direction reveals a flow connecting the central keyword ‘burial’ to ‘legislation,’ ‘public welfare,’ and ‘national land.’ This implies that funeral culture extends beyond a matter of individual choice and is closely linked to social and institutional domains, such as public policy, land use, and sanitation management.

Furthermore, the keywords ‘change’ and ‘paradigm’ are interpreted as reflecting the recent shift in funeral culture from a structure centered on traditional burial to one focused on cremation and eco-friendly funeral practices.

### 3.3. LDA Topic Modeling Analysis

LDA topic modeling was conducted to identify latent thematic structures within well-dying research. Since topic allocation varies depending on the selected number of topics, determining the optimal topic number is a critical process in topic modeling analysis.

**Figure 6:**
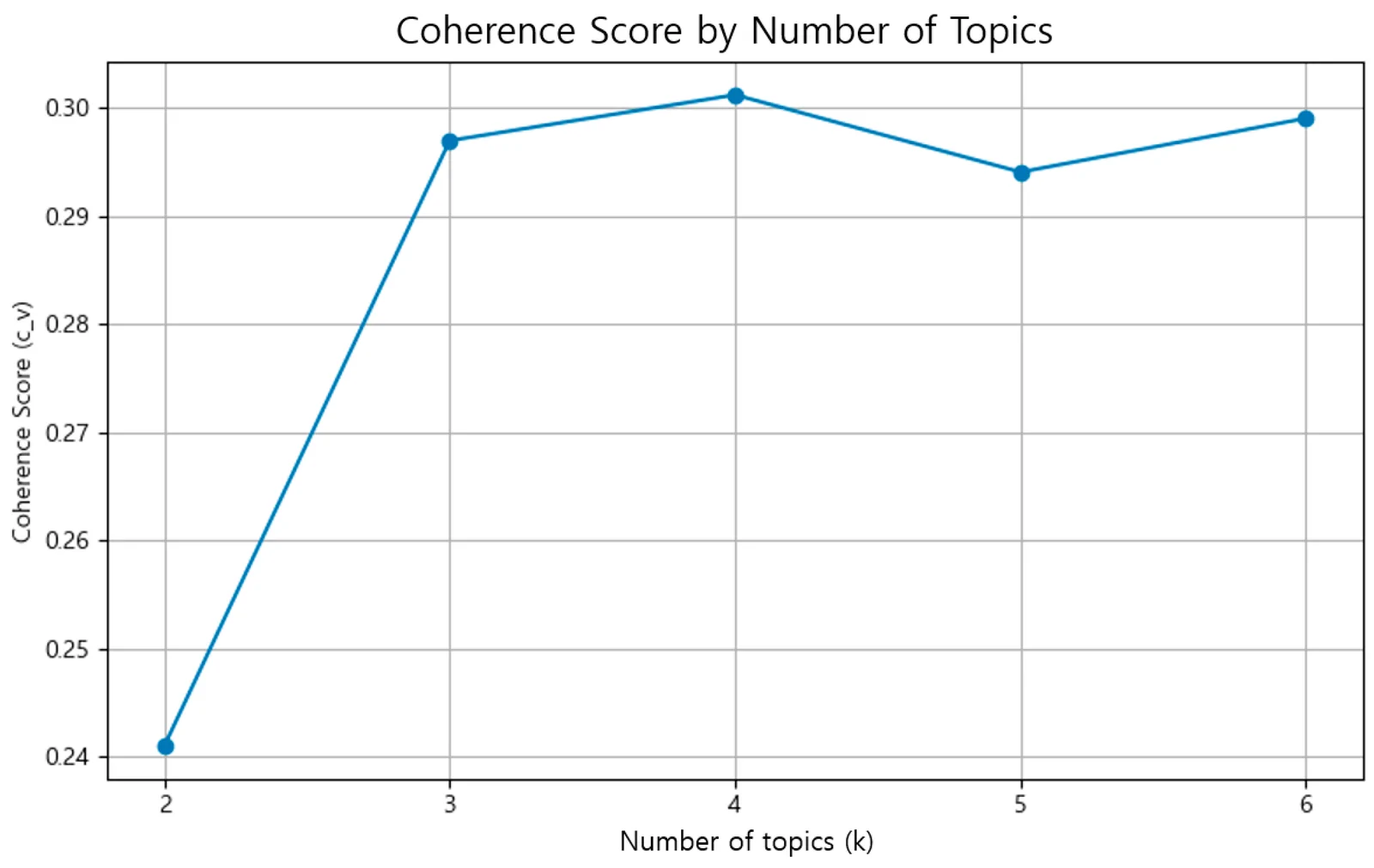
Coherence scores according to the number of topics.

In this study, topic coherence scores were repeatedly evaluated using the Gensim library in Python. During preprocessing, generic academic terms lacking semantic significance were removed as stopwords. As a result of repeatedly testing topic numbers ranging from two to six, the four-topic model achieved the highest coherence score (0.301). Although the coherence scores of the three- and four-topic models were relatively similar, the four-topic solution provided clearer thematic separation and greater interpretability in the Intertopic Distance Map generated using pyLDAvis. Moreover, the four topics corresponded well with the major semantic communities identified through the text network analysis. Therefore, the four-topic model was selected as the final solution because it achieved an appropriate balance between statistical performance and substantive interpretability. Figure 6 shows the distribution of coherence scores according to the number of topics.

The Intertopic Distance Map generated using pyLDAvis demonstrated that the four topics were relatively well separated with minimal overlap, indicating clear thematic distinctions among the extracted topics. Well-separated topic distributions generally indicate stronger topic independence and greater interpretability.

The topic modeling results identified four major themes within well-dying research.

**Figure 7:**
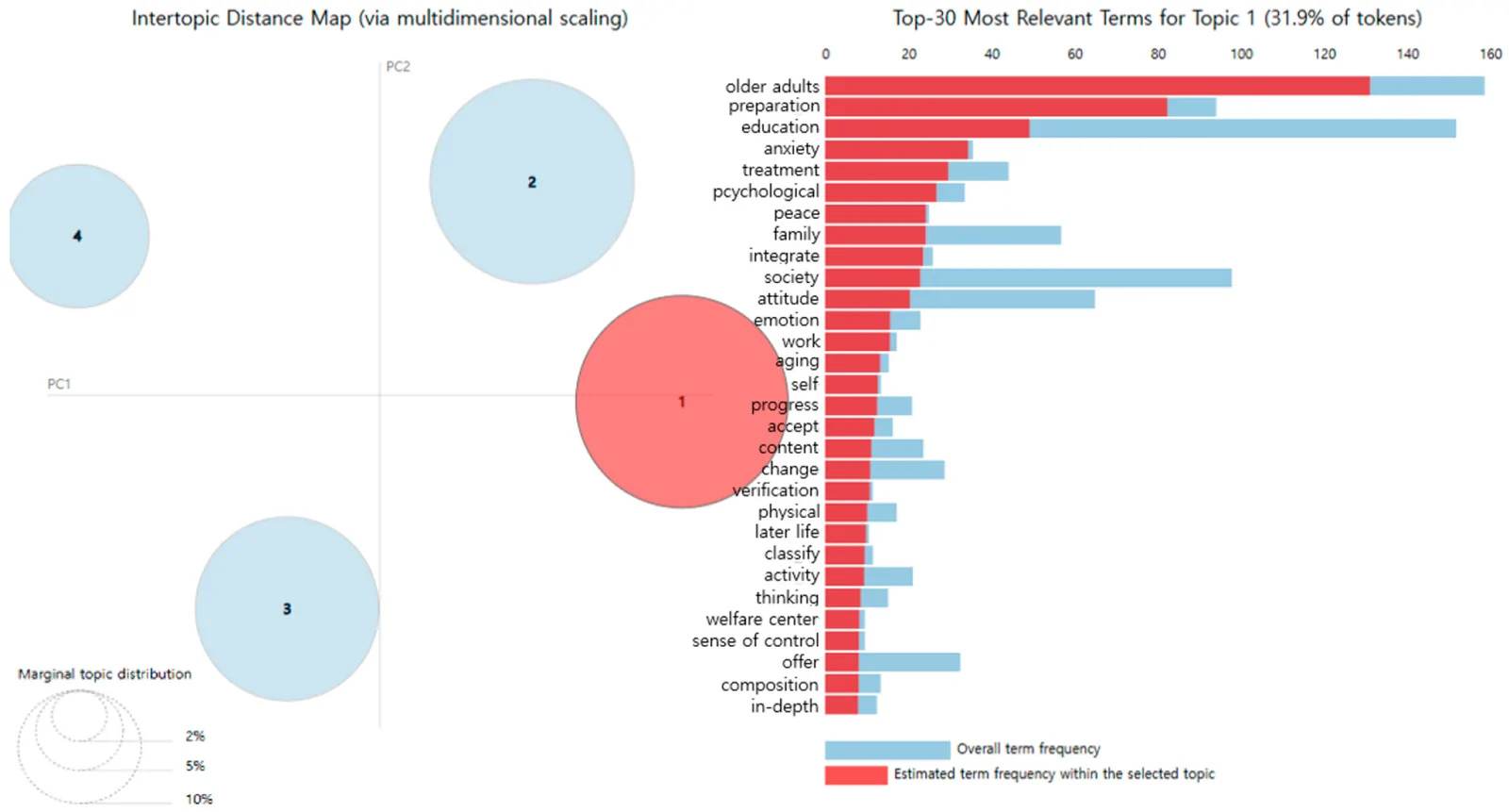
Distribution of keywords from Topic 1.

Figure 7 shows the word distribution of Topic 1.

- Topic 1: Psychological Well-Being and Death Preparation in Later Life

Topic 1 included keywords such as “older adults,” “preparation,” “education,” “anxiety,” “psychological well-being,” “family,” “self,” and “acceptance.” This topic reflects research related to death preparation, emotional adaptation, psychological stability, and acceptance of death in later life.

The results suggest that psychological support and death preparation education for older adults have become important components of well-dying research.

**Figure 8:**
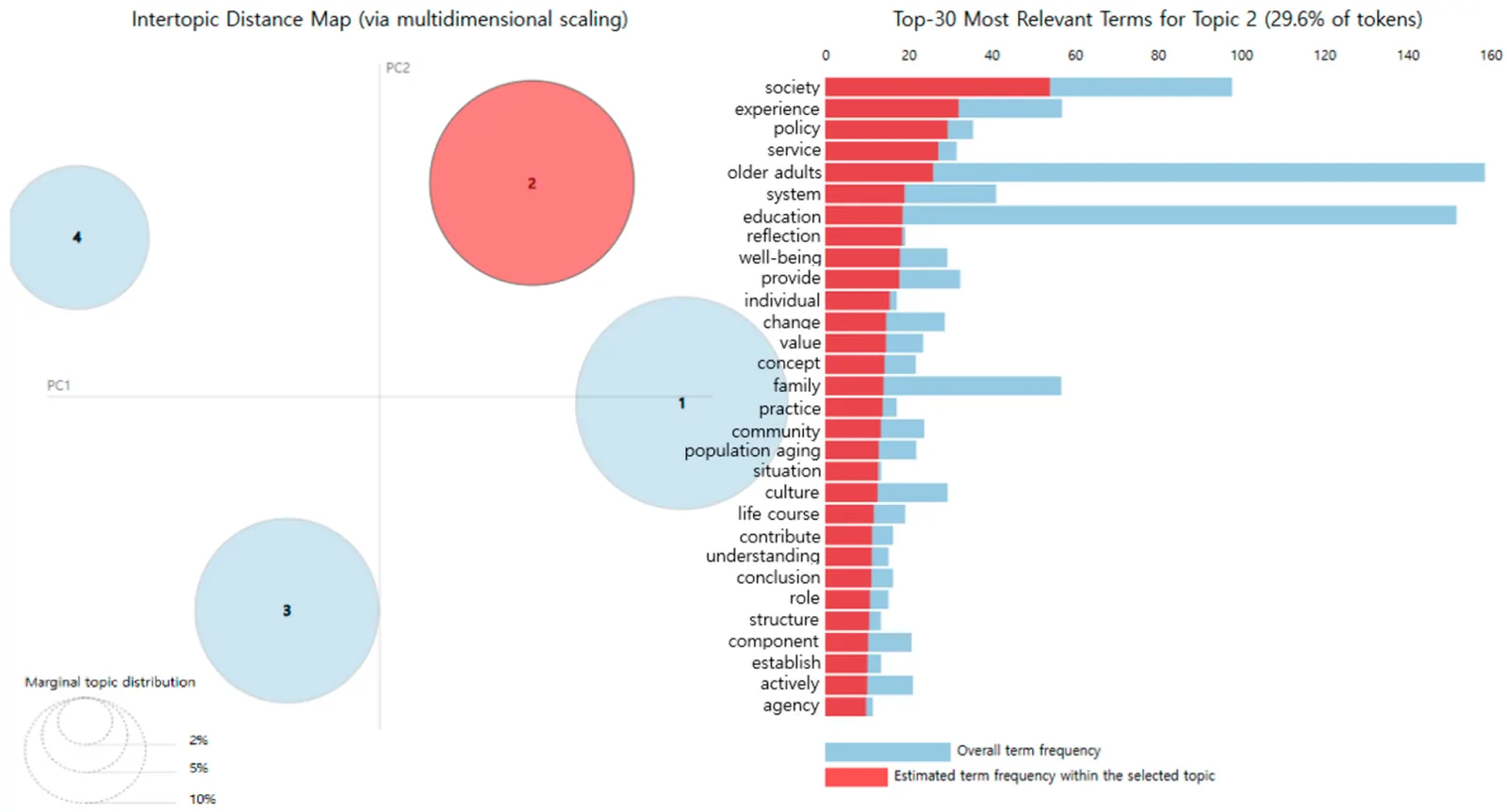
Keyword distribution in Topic 2.

Figure 8 shows the word distribution of Topic 2.

- Topic 2: Social and Policy Approaches to Well-Dying

Topic 2 consisted of keywords such as “society,” “policy,” “service,” “institution,” “well-being,” “community,” “population aging,” and “culture.” This topic reflects growing research interest in social support systems, community-based care, and policy responses for super-aged societies.

The findings indicate that well-dying is increasingly understood as a multidimensional social issue associated with welfare systems, public policy, and quality of life across the lifespan.

**Figure 9:**
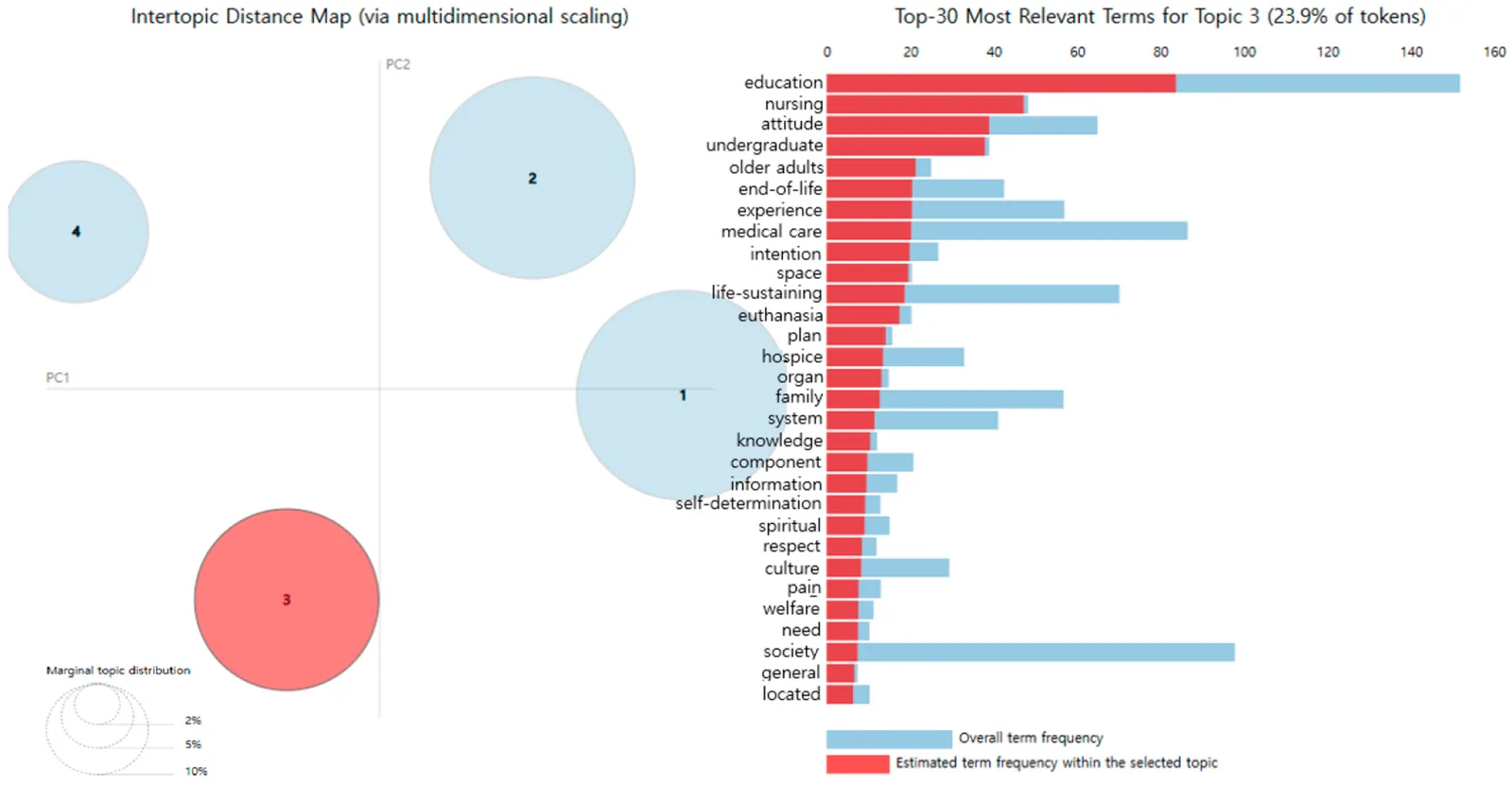
Keyword distribution in Topic 3.

Figure 9 shows the word distribution of Topic 3.

- Topic 3: Well-Dying Education and Healthcare Awareness

Topic 3 included keywords such as “education,” “nursing,” “medical care,” “hospice,” “self-determination,” “respect,” and “pain.” This topic reflects research on well-dying education, hospice and palliative care, and healthcare professionals’ awareness of end-of-life care.

The results indicate that educational interventions targeting healthcare professionals and nursing students have become increasingly important within the well-dying discourse.

**Figure 10:**
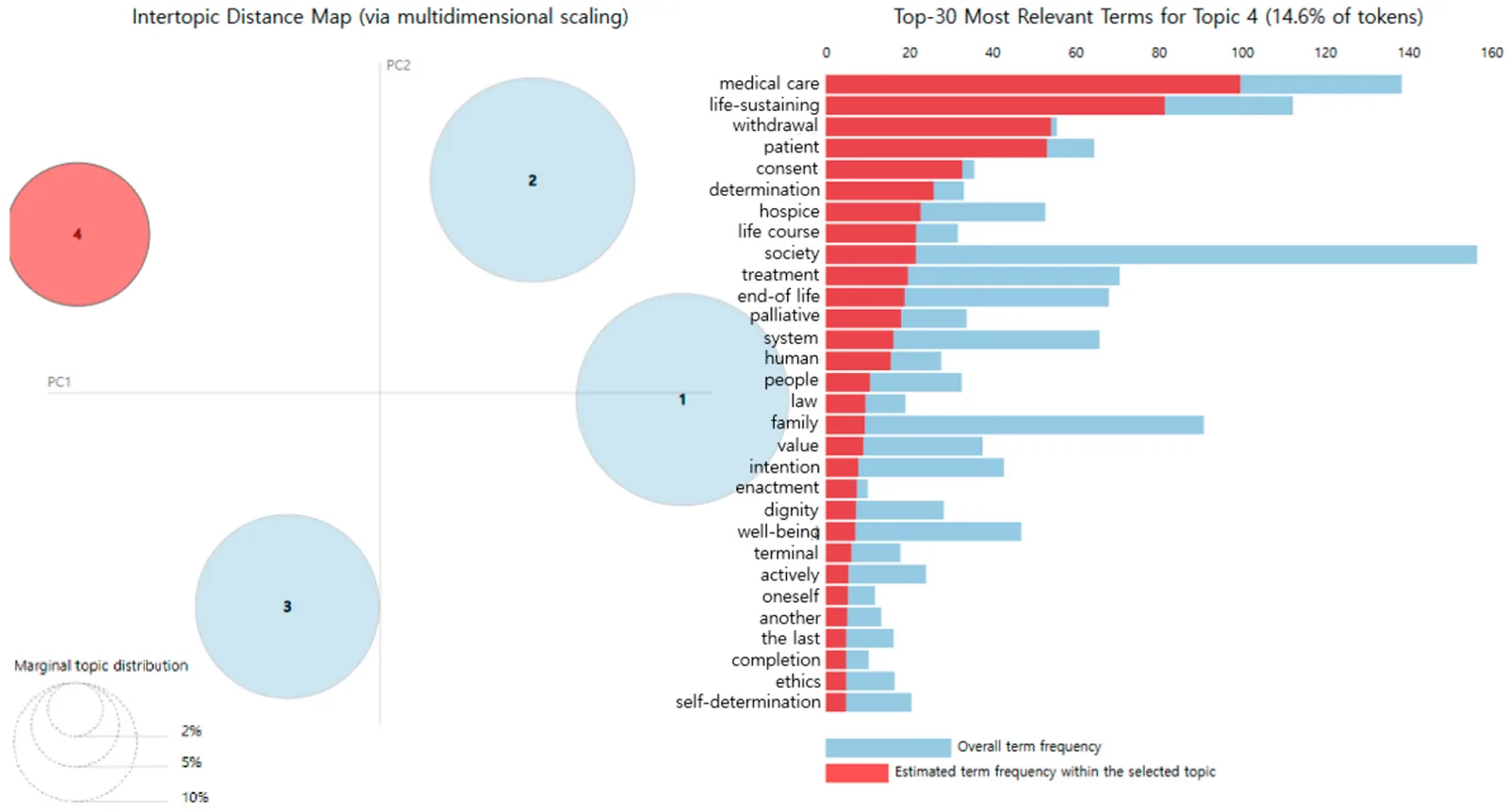
Keyword distribution in Topic 4.

Figure 10 shows the word distribution of Topic 4.

- Topic 4: Legal and Ethical Issues in Life-Sustaining Treatment Decisions

Topic 4 consisted of keywords such as “life-sustaining treatment,” “withdrawal,” “patient,” “consent,” “decision,” “legal,” “dignity,” and “self-determination.” This topic reflects legal and ethical discussions regarding end-of-life decision-making and patient autonomy.

The findings suggest that legal legitimacy, patient rights, and ethical decision-making related to life-sustaining treatment have emerged as major research themes following institutional reforms regarding end-of-life care.

Overall, the topic modeling results demonstrate that well-dying research in a rapidly aging society is structured around four interconnected domains involving psychological adaptation, healthcare education, legal and ethical decision-making, and community-based policy systems. These themes collectively illustrate the evolution of well-dying research from individual psychological preparation toward broader institutional and societal responses to population aging.

## 4. Discussion

This study investigated the knowledge structure and research trends of well-dying studies in the context of rapid population aging through text network analysis and topic modeling of 91 Korean academic studies published between 2016 and March 2026. The findings provide empirical evidence that the academic discourse surrounding well-dying has continuously evolved alongside demographic transition and changing societal demands. This evolution reflects the broader transformation of Korean society under rapid population aging and illustrates how academic research responds to emerging social and institutional challenges.

The findings indicate that well-dying research has evolved from early discussions focused primarily on death awareness and psychological acceptance toward broader multidimensional discussions involving healthcare, law, ethics, welfare systems, community-based support, and public policy [3,4]. This transition suggests that well-dying is increasingly recognized not merely as an issue of individual death preparation but as a multidimensional societal challenge requiring interdisciplinary responses. The expansion of research themes also reflects the growing recognition that achieving a good death depends not only on individual attitudes but also on healthcare systems, legal frameworks, community support, and public policy.

Keyword frequency analysis revealed that concepts such as “death,” “awareness,” “education,” “older adults,” “society,” “medical care,” and “life-sustaining treatment” occupied central positions within the research discourse. In particular, the emergence of keywords related to patient autonomy, legal decision-making, hospice care, and community welfare demonstrates that well-dying research increasingly reflects the institutional and social challenges associated with rapidly aging societies [2,5]. The increasing prominence of these institutional and policy-related keywords suggests that legislative reforms, expanding hospice and palliative care services, and healthy aging initiatives have substantially influenced the direction of well-dying research during the past decade.

Degree centrality analysis further demonstrated that concepts such as “death,” “awareness,” “education,” and “society” function as major connecting axes within the semantic network. The relatively balanced structure of the top keyword network suggests that well-dying research is not concentrated within a single academic domain but rather forms an interdisciplinary and convergent research field integrating psychology, healthcare, social welfare, ethics, and policy studies [2]. This interdisciplinary structure indicates that well-dying has become an integrative research domain requiring collaboration among healthcare professionals, social scientists, educators, legal scholars, and policymakers to address increasingly complex end-of-life issues.

The community modularity analysis revealed several meaningful substructures involving life-sustaining treatment decisions, legal and institutional systems, pain alleviation, dignity, and community-based responses to aging societies [18,19]. In particular, the relatively high modularity values indicate that well-dying research forms clearly distinguishable thematic clusters. Furthermore, the identification of independent clusters related to funeral culture and memorial systems suggests that well-dying research is increasingly extending beyond end-of-life medical care to encompass post-death socio-cultural processes. These findings indicate that well-dying is increasingly understood as a continuum encompassing preparation for death, end-of-life care, bereavement, and broader socio-cultural processes rather than as an isolated medical event.

Topic modeling analysis identified four major themes: psychological well-being and death preparation in later life; social and policy approaches to well-dying; well-dying education and healthcare awareness; and legal and ethical issues surrounding life-sustaining treatment decisions. These findings suggest that well-dying research has gradually evolved from individual psychological adaptation toward broader institutional and policy-oriented approaches. Taken together, the four topics demonstrate that contemporary well-dying research increasingly integrates healthcare, legal systems, ethics, education, and social policy, reflecting the complexity of supporting quality of life and dignity throughout the end-of-life process.

Importantly, the overall research structure identified in this study reflects the growing societal need for integrated end-of-life support systems in super-aged societies. The transition from psychological preparation and death awareness toward legal systems, healthcare decision-making, community care, and public policy demonstrates that well-dying is increasingly recognized as a major social issue rather than solely an individual concern [20]. These findings support the conceptual framework proposed in this study, suggesting that demographic transition not only changes patterns of aging but also reshapes academic priorities, institutional responses, and public perceptions regarding well-dying. Consequently, well-dying should be understood as an essential component of healthy aging and sustainable community care rather than solely as an issue associated with the final stage of life.

As one of the world’s fastest-aging societies, South Korea provides an important early case for understanding how well-dying research evolves under conditions of accelerated demographic aging [1]. The findings of this study therefore provide meaningful implications not only for Korea but also for other countries preparing for similar demographic transitions and increasing demands for end-of-life care and community support systems. Countries currently experiencing rapid population aging may encounter similar shifts in research priorities and policy development, making the Korean experience a useful reference for comparative studies and international discussions on healthy aging and integrated end-of-life care.

This study is academically significant because it systematically analyzed the intellectual structure and thematic evolution of well-dying research by integrating text network analysis and topic modeling. Unlike previous studies that focused on specific issues or individual empirical cases, this study demonstrated that well-dying research forms a multidimensional and interdisciplinary knowledge structure involving healthcare, psychology, law, ethics, welfare, and public policy. Methodologically, this study also demonstrates the value of integrating text network analysis and topic modeling to examine the evolution of academic knowledge structures, providing a complementary approach to conventional narrative literature reviews.

Nevertheless, several limitations should be acknowledged. First, this study analyzed only KCI-indexed Korean academic publications, which was appropriate for investigating the evolution of well-dying research within Korea but may limit the generalizability of the findings to other cultural and institutional contexts. Second, because this study primarily relied on keyword-based semantic relationships, deeper contextual meanings embedded within individual articles may not have been fully captured. Future research should therefore incorporate comparative international analyses using multiple academic databases and combine computational text analysis with qualitative approaches to provide a more comprehensive understanding of the cultural and institutional diversity of well-dying research.

## 5. Conclusions

This study analyzed the major research trends and knowledge structures of well-dying studies in the context of rapid population aging through text network analysis and topic modeling.

The findings revealed that well-dying research is centered on themes such as death awareness, psychological adaptation, healthcare decision-making, hospice care, self-determination, and community-based support systems. In particular, concepts related to life-sustaining treatment decisions, legal and ethical issues, dignity, and public policy have emerged as increasingly important research domains.

Community modularity analysis demonstrated that well-dying research forms several interconnected thematic structures involving healthcare, law, ethics, welfare systems, and socio-cultural responses to aging societies. In addition, topic modeling identified four major thematic domains involving psychological well-being, policy systems, healthcare education, and legal and ethical decision-making.

Overall, the findings indicate that well-dying research in rapidly aging societies is evolving from individual-centered death preparation toward broader multidimensional approaches involving healthcare systems, institutional frameworks, welfare policies, and community support structures.

As one of the world’s fastest-aging societies, the Korean case provides meaningful implications for countries preparing for similar demographic transitions. The results highlight the importance of integrated well-dying policies, hospice and palliative care systems, community-based support networks, and interdisciplinary approaches to end-of-life care in super-aged societies.

Therefore, future well-dying research should continue expanding beyond medical and legal perspectives to include psychological well-being, community care, cultural values, funeral and memorial practices, and life-course approaches. In addition, practical discussions regarding integrated end-of-life support systems and aging policies should continue to improve quality of life and dignity in rapidly aging societies.

## Figures and Tables

**Table 1:** Top 30 keywords and their frequencies.

No.	Keyword	Frequency	No.	Keyword	Frequency
1	death	351	16	patient	44
2	awareness	207	17	system	43
3	education	164	18	undergraduate	41
4	older adults	160	19	withdrawal	38
5	society	102	20	anxiety	36
6	preparation	97	21	policy	36
7	medical care	95	22	hospice	35
8	law	89	23	psychological	34
9	life-sustaining	77	24	service	33
10	attitude	68	25	decision	33
11	experience	59	26	well-being	32
12	family	59	27	culture	31
13	nursing	51	28	change	31
14	treatment	46	29	welfare	31
15	end-of-life	45	30	integration	30

**Table 2:** Distribution of degree centrality scores.

Measures	131 Keywords	30 Keywords
Mean	0.597	0.897
Std.Dev	0.189	0.111
Min.	0.046	0.552
Network Degree Centralization Index	0.4093	0.1108

**Table 3:** Degree centrality and community of the top 20% of core keywords.

No.	Keyword	Degree Centrality	Community
1	aging	0.85	1
2	family	0.845	1
3	medical care	0.815	1
4	decision	0.79	1
5	law	0.78	1
6	patient	0.745	1
7	system	0.725	1
8	community	0.7	1
9	end-of-life	0.7	1
10	policy	0.675	1
11	culture	0.675	1
12	awareness	0.955	2
13	preparation	0.9	2
14	quality of life	0.885	2
15	life course	0.98	3
16	death	0.98	3
17	society	0.955	3
18	education	0.925	3
19	older adults	0.865	3
20	experience	0.815	3
21	attitude	0.775	3
22	change	0.765	3
23	treatment	0.695	3
24	psychological	0.685	3
25	dignity	0.675	3

## Data Availability

The bibliographic data analyzed in this study were obtained from the Korean Citation Index (KCI), a publicly accessible academic database. The data supporting the findings of this study are included in the article and Appendix A. Additional information regarding the dataset used for the analysis is available from the corresponding author upon reasonable request.

## Appendix A. Literature Search Strategy

**Table A1:** Summary of the literature search strategy.

Item	Description
Database	Korean Citation Index (KCI)
Database URL	https://www.kci.go.kr/
Search period	January 2016—March 2026
Literature retrieval date	1 April 2026
Search fields	Korean title; English author keywords
Korean search term	“웰다잉”
English search term	“well-dying”
Inclusion criteria	Peer-reviewed KCI-indexed journal articles focusing primarily on well-dying
Exclusion criteria	Duplicates, non-journal publications, studies not primarily focused on well-dying
Search query	Korean title = “웰다잉” AND English author keyword = “well-dying”
Final dataset	91 articles
